# Neuroprotective potential of quetiapine against streptozotocin‐induced sporadic Alzheimer’s disease

**DOI:** 10.1002/alz.088739

**Published:** 2025-01-03

**Authors:** Shilpa Kumari, Paras Attri, Rahul Renukadas Deshmukh

**Affiliations:** ^1^ Maharaja Ranjit Singh Punjab Technical University, Bathinda, Punjab India; ^2^ Mahraja Ranjit Singh Punjab Technical University,, Bathinda, Bathinda India; ^3^ Department of Pharmacology, Central University of Punjab, Bathinda, Bathinda, Punjab India

## Abstract

**Background:**

In previous studies, we found that quetiapine activates the AKT signaling which further inhibits the action of GSK3β. Quetiapine has been reported to possess neuroprotective potential in schizophrenia and other neurodegenerative models.

**Method:**

On day 1^st^ and 3^rd^, rats received bilateral intracerebroventricular (i.c.v.) administration of STZ (3 mg/kg). From day 8 to day 21 of treatment of quetiapine, a GSK3‐β inhibitor and neuroprotective medication, was given at dose levels of 2.5, 5, and 10 mg/kg/i.p. Morris water maze and Object recognition test was performed for the evaluation of cognitive impairment, whereas biochemical, histopathological, neuroinflammatory were evaluated by hippocampal and cortical brain region on 22^nd^ day.

**Result:**

ICV‐STZ results in cognitive impairment, spatial and non‐special learning deficit in OFT, MWM and ORT tests respectively, including elevation in the levels of nitrite and MDA and decreased antioxidant enzymes GSH, SOD and Catalase levels, also produced degenerative changes in dentate gyrus, CA1, CA2 and CA3 region of hippocampus as well as cortex in rat brains. Whereas, Quetiapine treatment significantly attenuated STZ‐induced cognitive deficit, oxidative stress and improved density of surviving neurons in hippocampal/cortical region of rat brains.

**Conclusion:**

The conclusive outcomes of the present study, clearly indicate neuroprotective potential of quetiapine in neurodegenerative pathogenesis associated with cognitive dysfunction.

